# Biotransformation of Pesticides across Biological Systems: Molecular Mechanisms, Omics Insights, and Biotechnological Advances for Environmental Sustainability

**DOI:** 10.1021/acsomega.5c06484

**Published:** 2025-10-24

**Authors:** Gayatri Basapuram, Avishek Dutta, Srimanti Duttagupta

**Affiliations:** † Department of Geology, 1355 University of Georgia, Athens, Georgia 30602, United States; ‡ Savannah River Ecology Laboratory, University of Georgia, Aiken, South Carolina 29802, United States

## Abstract

The widespread application of pesticides such as organophosphates, organochlorides, and triazines in modern agriculture has led to their notable presence in soils, water bodies, and food chains, raising concerns about persistence, bioaccumulation, and adverse effects on nontarget organisms. Biotransformation, the enzymatic transformation of xenobiotic compounds by microorganisms, plants, and animals, plays a pivotal role in the degradation and detoxification of these chemicals. This review provides a comprehensive examination of the mechanisms, key enzyme classes (e.g., hydrolases, oxidoreductases, transferases), and environmental factors influencing pesticide biotransformation across different biological systems. Recent advances in omics technologies have revolutionized the understanding of microbial and plant metabolism, while synthetic biology offers opportunities for engineering enhanced degradation capabilities. The environmental fate of transformation products is also discussed, together with a critical analysis of challenges, unresolved questions, and future research directions, offering a holistic perspective on pesticide biotransformation as a key process for mitigating chemical pollution.

## Graphical abstract

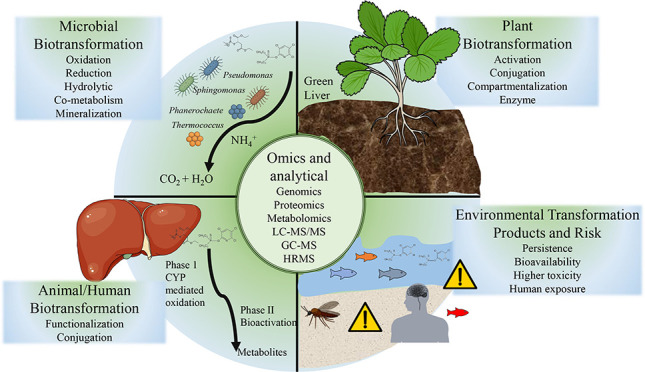

## 1 Introduction

Pesticides, including herbicides, insecticides, fungicides, and nematicides, have been instrumental in securing agricultural productivity and public health.^1^ Their global consumption exceeded 4 million tons annually by the early 2020s.^2^ While their application has ensured food security and vector control, the environmental burden posed by their residues is substantial. Pesticide contamination has been reported in diverse matrices of soils, surface waters, groundwater aquifers, and even atmospheric aerosols, owing to their chemical stability, mobility, and persistence.^3,4^ Compounds such as organochlorines and triazine herbicides are notorious for their long half-lives and tendency to bioaccumulate, leading to biomagnification in food webs.^5,6^ Beyond their intended effects, pesticides exert sublethal and chronic toxicities on nontarget organisms, disrupt ecosystem services, and threaten biodiversity.^7,8^ Consequently, mitigating pesticide pollution is a global priority.

Biotransformation refers to the biochemical modification of chemical compounds by living organisms and serves as a fundamental process for the natural attenuation of environmental contaminants, including pesticides.^9–11^ Through a variety of enzymatic mechanisms, biotransformation converts hydrophobic and chemically persistent pesticides into more hydrophilic and often less toxic metabolites, thereby enhancing their solubility, bioavailability, and eventual elimination from the environment.^12,13^ This process is facilitated by a diverse array of organisms, including microorganisms, plants, and animals, each employing specialized enzymatic systems tailored to their ecological niches. In microbial systems, biotransformation pathways often culminate in complete mineralization, where pesticides are broken down into inorganic constituents such as carbon dioxide, water, and ammonia.^14,15^ In contrast, plants and animals primarily aim to detoxify and sequester pesticides rather than achieve complete mineralization. Plants metabolize pesticides through a series of enzymatic reactions that mirror the xenobiotic metabolism observed in animals, often described as the “green liver” model.^16,17^ In animals, particularly mammals, pesticide biotransformation occurs predominantly in the liver via Phase I functionalization and Phase II conjugation reactions, which render the compounds more amenable to excretion.^18,19^ However, biotransformation is not invariably synonymous with detoxification. Certain metabolic intermediates may exhibit greater toxicity or environmental persistence than their parent compounds. For instance, the cytochrome P450-mediated oxidative metabolism of organophosphorus pesticides, such as chlorpyrifos, leads to the formation of oxon derivatives, which are potent acetylcholinesterase inhibitors and thus present significant neurotoxic risks to nontarget organisms, including humans.^20,21^ This paradox highlights the complexity of biotransformation pathways and underscores the necessity for comprehensive environmental risk assessments that consider both parent compounds and their metabolites. The environmental half-lives of pesticides are highly variable, ranging from a few days to several years depending on intrinsic chemical properties such as molecular structure and extrinsic environmental factors including temperature, pH, and the composition of microbial communities.^22–24^ Without efficient biotransformation, pesticides can persist in ecosystems, resulting in chronic exposure, biomagnification, and subsequent adverse effects on wildlife and human health.^25–27^ Microorganisms play a particularly significant role in the natural attenuation of pesticide residues, especially in soil and aquatic environments, where they exploit pesticides either as a sole carbon and energy source or cometabolically during the degradation of other substrates.^28^ Phytoremediation, which leverages the metabolic capabilities of plants, offers an environmentally sustainable and cost-effective strategy for the decontamination of pesticide-impacted soils and water bodies.^29,30^ In animals, biotransformation pathways mitigate the bioaccumulation of pesticides, although interspecies variability in metabolic capacity can influence differential susceptibility to pesticide toxicity.^31^ Recent advancements in high-throughput omics technologies including genomics, transcriptomics, proteomics, and metabolomics have significantly expanded the understanding of the molecular underpinnings of biotransformation.^31^ These technologies have facilitated the identification of novel degradation pathways, key enzymatic players, and regulatory networks, offering new opportunities for the development of genetically engineered microorganisms and plants with enhanced bioremediation capabilities.^32–34^

This review offers a comprehensive synthesis of current knowledge regarding the biotransformation of pesticides, encompassing the pathways employed by microorganisms, plants, and animals. Central to this discussion is an examination of the key enzymatic systems and regulatory mechanisms that mediate the breakdown of pesticide molecules. Advances in omics technologies and analytical methodologies that have significantly deepened the understanding of these processes are explored. Specific attention is given to the environmental fate of transformation products, which often exhibit distinct behaviors and toxicities compared to their parent compounds. In this review, “biotransformation” refers to enzymatic modifications of pesticides, “biodegradation” to microbial breakdown often leading to mineralization, and “detoxification” to metabolic conversions that reduce toxicity without necessarily achieving complete degradation. The review further addresses integrated remediation strategies that combine biological, chemical, and technological approaches to enhance the degradation and removal of pesticide residues from contaminated environments. Additionally, it identifies prevailing knowledge gaps and delineates future research directions essential for refining the understanding of biotransformation processes. Through this synthesis, the review aims to advance a holistic perspective on pesticide biotransformation that informs the development of sustainable management practices and regulatory frameworks for mitigating the environmental and health impacts of pesticide use.

## 2 Pesticide Biotransformation Pathways

### 2.1 Overview of Biotransformation Mechanisms

Biotransformation processes can be categorized into two phases. Phase I reactions involve functionalization (e.g., oxidation, reduction, hydrolysis), introducing or exposing functional groups on the pesticide molecule.^35^ Phase II reactions involve conjugation with endogenous molecules (e.g., glutathione, sulfate, glucuronic acid), increasing solubility and facilitating excretion.^36^ Phase I reactions are primarily catalyzed by cytochrome P450 monooxygenases, carboxylesterases, and peroxidases. These reactions generally reduce the toxicity of pesticides but can sometimes produce more toxic intermediates.^37^ Phase II reactions conjugate the functionalized pesticides with polar molecules, mediated by transferases such as glutathione S-transferases, UDP-glucuronosyltransferases, and sulfotransferases. The conjugated metabolites are typically less toxic and more water-soluble^38^ (Table S1).

### 2.2 Plant Metabolic Detoxification Pathways

Plants have developed sophisticated biochemical defense mechanisms to cope with a wide array of xenobiotic substances, including pesticides^39^ (Table S2). Unlike animals that can metabolize xenobiotics in specific detoxifying organs like the liver, plants perform detoxification at the cellular level, distributing the metabolic burden across their tissues.^40^ The detoxification of pesticides in plants is traditionally described as a three-phase process, often referred to as the “green liver” model, analogous to the liver metabolism in animals.^41^

#### 2.2.1 Phase I: Activation

The first phase of pesticide detoxification in plants involves the activation of the pesticide molecule by introducing or exposing functional groups through oxidation, reduction, or hydrolysis reactions. This phase is primarily catalyzed by oxidative enzymes such as cytochrome P450 monooxygenases (CYPs), peroxidases, and esterases.^42,43^

##### 2.2.1.1 Cytochrome P450 Monooxygenases

CYPs are heme-thiolate proteins that catalyze the insertion of an oxygen atom into the pesticide substrate (RH + O_2_ + NADPH → ROH + H_2_O). This reaction typically results in hydroxylation, epoxidation, or dealkylation, enhancing the reactivity of the pesticide molecule and preparing it for subsequent conjugation reactions. CYP450 enzymes are highly versatile, capable of metabolizing a wide spectrum of chemical structures, and their expression can be induced by exposure to pesticides.^44^

##### 2.2.1.2 Peroxidases

These enzymes catalyze the oxidation of a broad range of substrates using hydrogen peroxide as the oxidant. Peroxidases are involved in lignin biosynthesis and also participate in the oxidative degradation of pesticides.^45^

##### 2.2.1.3 Esterases and Hydrolases

Esterases cleave ester bonds commonly found in herbicides and insecticides, producing more polar metabolites. This hydrolytic cleavage is crucial for herbicides like atrazine and carbamates.^46^ The activation phase increases the hydrophilicity and reactivity of the pesticides, enabling further metabolism in the subsequent phase.^43^

#### 2.2.2 Phase II: Conjugation

In Phase II, the activated pesticide metabolites are conjugated with endogenous hydrophilic molecules, rendering them less toxic and more water-soluble.^47^ Major conjugation reactions include:

##### 2.2.2.1 Glutathione Conjugation

Glutathione S-transferases (GSTs) catalyze the conjugation of glutathione (GSH) to electrophilic centers on the activated pesticide molecules.^48^ This reaction detoxifies harmful compounds and aids in their sequestration or transport within the plant cells. Glutathione conjugation is crucial for herbicides such as atrazine and alachlor.^49^

##### 2.2.2.2 Glycosylation

UDP-glucosyltransferases (UGTs) mediate the attachment of sugar moieties (primarily glucose) to hydroxylated or carboxylated pesticide metabolites. This glycosylation increases the molecular size and water solubility, facilitating storage in the vacuole.^50^

##### 2.2.2.3 Amino Acid Conjugation

Some pesticides are conjugated with amino acids such as glutamate, aspartate, or alanine. The herbicide 2,4-dichlorophenoxyacetic acid (2,4-D) can be conjugated with aspartate in plants.^51^

##### 2.2.2.4 Methylation

Although less common, methyltransferases can methylate hydroxyl groups on pesticide metabolites, leading to further detoxification.^52^

These conjugation reactions not only detoxify the pesticides but also prepare them for sequestration, reducing their phytotoxic effects.

#### 2.2.3 Phase III: Compartmentalization

The final phase of plant pesticide detoxification involves the sequestration of conjugated metabolites into vacuoles or their binding to cell wall components. This phase effectively immobilizes pesticide residues, preventing their interference with cellular processes.

##### 2.2.3.1 Vacuolar Sequestration

Transporters, primarily from the ATP-binding cassette (ABC) transporter family, are responsible for the active transport of conjugated metabolites into vacuoles. Sequestration into vacuoles isolates the metabolites from the cytoplasm, mitigating their potential toxicity.^53^

##### 2.2.3.2 Cell Wall Binding

In some cases, conjugated pesticide metabolites can become covalently bound to lignin or other cell wall polymers. This binding is considered irreversible and serves as a long-term detoxification mechanism.^54^

Phase III is crucial for the long-term management of xenobiotic compounds within the plant, particularly in perennial species where metabolites may persist over growing seasons.

#### 2.2.4 Enzymatic Systems Involved

Plant detoxification relies on a complex array of enzymes that are highly inducible upon pesticide exposure:

##### 2.2.4.1 Cytochrome P450 Monooxygenases (CYPs)

Over 200 CYP genes have been identified in *Arabidopsis thaliana* alone, with several families (e.g., CYP71, CYP72) involved in xenobiotic metabolism.^55^

##### 2.2.4.2 Glutathione S-Transferases (GSTs)

Plant GSTs are categorized into several classes, including phi (F), tau (U), and theta (T), with phi and tau classes being most prominent in detoxification.^56^

##### 2.2.4.3 UDP-Glucosyltransferases (UGTs)

UGTs belong to a large gene family responsible for glycosylation reactions. They exhibit substrate specificity but often display overlapping functions, providing redundancy and flexibility in detoxification pathways.

## 3 Mechanisms of Microbial Pesticide Degradation

Microbial pesticide degradation is a central component of natural attenuation processes in contaminated environments. This capability stems from the remarkable metabolic plasticity of microorganisms, enabling them to transform and mineralize complex xenobiotic compounds.^57^ The degradation mechanisms can be broadly categorized into four primary pathways: oxidative transformations, reductive transformations, hydrolytic cleavage, and cometabolic processes. Each mechanism involves distinct biochemical strategies and is influenced by both intrinsic microbial factors and extrinsic environmental conditions.^58^ Understanding these mechanisms is pivotal for designing effective bioremediation strategies and for predicting the environmental fate of pesticides (Table S1 and Figure S1–S3).

### 3.1 Oxidative Transformations

Oxidative processes are the most common microbial degradation pathways, particularly under aerobic conditions. These reactions often constitute the initial step in pesticide breakdown, making compounds more polar and susceptible to further metabolism. Monooxygenases catalyze the insertion of a single oxygen atom into the substrate molecule, utilizing cofactors such as NADH, NADPH, and flavin adenine dinucleotide (FAD). These enzymes belong to the larger class of oxidoreductases and include cytochrome P450 monooxygenases, flavin-containing monooxygenases, and molybdenum-containing oxidases.^59^ For instance, cytochrome P450 monooxygenases (CYP450s) introduce hydroxyl groups into the pesticide structure, a process critical for the initial activation of hydrophobic molecules.^60^ This is exemplified in the oxidation of the triazine herbicide atrazine to hydroxylated metabolites by Pseudomonas spp..^61,62^ Dioxygenases differ from monooxygenases by incorporating both atoms of molecular oxygen into substrates. They are pivotal in the ring cleavage of aromatic compounds, leading to the breakdown of otherwise persistent structures. Catechol 2,3-dioxygenase cleaves catechol intermediates in the ortho- or meta-position during the degradation of phenolic pesticide derivatives.^63^ In fungi, oxidative degradation is facilitated by extracellular lignin-modifying enzymes such as lignin peroxidase (LiP), manganese peroxidase (MnP), and laccases.^64^ These enzymes are nonspecific oxidizers and are particularly effective against persistent organic pollutants, including organochlorine pesticides.^65^

### 3.2 Reductive Transformations

Reductive reactions are prevalent under anaerobic conditions and are particularly important for the degradation of halogenated pesticides, which are highly recalcitrant due to the stability imparted by halogen atoms. In reductive dehalogenation, microorganisms catalyze the sequential removal of halogen atoms, typically chlorine, from the pesticide molecule, often utilizing reductive dehalogenase enzymes.^66^ This process reduces the electronegativity of the compound, enhancing its susceptibility to further degradation.^67^ Dehalobacter and Dehalococcoides species have been shown to reductively dechlorinate hexachlorocyclohexane (HCH) isomers and DDT, leading to less chlorinated and more biodegradable products such as monochlorobenzene and benzene.^68^ The mechanism generally involves enzymatic transfer of electrons to the pesticide, stepwise elimination of halogen atoms, and generation of less recalcitrant compounds. Certain microbial species can reduce nitro groups in pesticides such as nitrophenols and trinitrotoluene (TNT) to corresponding amines, further facilitating degradation.^69^

### 3.3 Hydrolytic Degradation

Hydrolysis is a prominent microbial degradation pathway for pesticides containing ester, amide, carbamate, and phosphoric acid ester bonds. Hydrolytic enzymes cleave these bonds by adding a water molecule, leading to the formation of more hydrophilic and less toxic products. Esterases hydrolyze ester bonds; amidases cleave amide bonds; and phosphotriesterases target organophosphate ester linkages. These enzymes are commonly found in soil-dwelling bacteria such as Pseudomonas and Bacillus sp..^70^ Carbamate insecticides like carbaryl are degraded by carbaryl hydrolase, an enzyme capable of cleaving the *N*-methyl carbamate bond to yield 1-naphthol and methylamine.^71^ Organophosphate pesticides such as parathion and malathion undergo hydrolysis by phosphotriesterases, resulting in less toxic diethyl phosphates and thiols.^72^ Hydrolytic degradation is critical because many modern pesticides, including pyrethroids and neonicotinoids, possess ester or amide linkages susceptible to microbial hydrolysis.^73^

### 3.4 Co-Metabolism

Co-metabolism is a phenomenon where microorganisms transform into pesticide incidentally while metabolizing a primary growth substrate. Importantly, the pesticide does not support microbial growth or provide energy; rather, its degradation is a side reaction catalyzed by enzymes induced by the growth substrate. Methanotrophs (methane-oxidizing bacteria) cometabolically degrade halogenated pesticides and solvents via methane monooxygenase (MMO) enzymes.^74^ Trichloroethylene (TCE) and chloroform are degraded during methane oxidation by Methylosinus trichosporium.^75^ Carbofuran degradation by Pseudomonas sp., during glucose metabolism has also been observed.^76^ Co-metabolism is crucial for the breakdown of highly recalcitrant pesticides that resist direct metabolism, such as DDT and atrazine.^77^

### 3.5 Mineralization

Mineralization refers to the complete degradation of the pesticide to inorganic compounds like CO_2_, NH_4_^+^, and H_2_O.^78^ True mineralization is often the goal of bioremediation because it ensures no persistent metabolites remain. Pathways leading to mineralization generally require a consortium of microorganisms, each capable of degrading intermediates generated by others.^79^ The mineralization of atrazine to CO_2_ and NH_4_^+^ involves several steps and microbial species, each catalyzing specific reactions such as dechlorination, deamination, and ring cleavage.

Several factors affect the efficiency and pathway of microbial pesticide degradation, such as pesticide concentration: high concentrations may inhibit microbial activity due to toxicity, adsorption of pesticides to soil particles can limit microbial access, environmental conditions such as oxygen levels, pH, temperature, and nutrient availability play significant roles, microbial community structure and diversity and functional redundancy enhance degradation capacity, cosubstrates and cocontaminants, presence of easily degradable organics can promote cometabolic pathways.^80^ Advanced bioreactor designs and bioaugmentation strategies often aim to optimize these factors to maximize degradation efficiency.^81^

Representative enzymes demonstrate characteristic pH and temperature optima that govern pesticide degradation efficiency. Organophosphorus hydrolases (OPHs/phosphotriesterases) generally perform best under alkaline conditions (pH ∼ 8–10), with thermophilic variants such as OPHC2 retaining activity up to ∼65 °C.^82^ Laccases used in oxidative degradation typically exhibit acidic optima (pH 2–6) and function across 40–70 °C, depending on their source or immobilization strategy.^83^ Similarly, pyrethroid-degrading esterases such as Est804 show highest activity under alkaline pH (≈8–10) at 35–50 °C, while immobilized variants (e.g., Est882) maintain stability from pH 8–11 and remain active around 37 °C.^84^

## 4 Key Microbial Taxa in Pesticide Biotransformation

Microbial degradation of pesticides is mediated by a wide variety of microorganisms inhabiting diverse ecological niches, ranging from soil to aquatic environments.^85^ The capability to biotransform and mineralize pesticides is not confined to a few specialized species but is rather distributed across multiple taxonomic groups. This metabolic versatility is an outcome of evolutionary adaptations to xenobiotic pressures and has been further augmented by horizontal gene transfer mechanisms, which facilitate the dissemination of degradative traits across microbial populations.^86^ The microbial taxa involved in pesticide biotransformation encompass a broad phylogenetic range, including bacteria, fungi, and archaea, each contributing uniquely to the degradation processes.^87,88^ Bacteria constitute the most extensively studied group in pesticide biotransformation due to their metabolic diversity, rapid growth rates, and adaptability to environmental fluctuations. Within the bacterial domain, the genus Pseudomonas has emerged as a model for pesticide degradation studies.^89^ Different strains of species such as *Pseudomonas putida*, *Pseudomonas fluorescens*, and *Pseudomonas stutzeri* possess a remarkable repertoire of catabolic enzymes capable of degrading a wide spectrum of pesticides, including organophosphates, carbamates, and chlorinated herbicides.^90,91^ These bacteria employ a suite of oxidative and hydrolytic enzymes, such as oxygenases, esterases, and hydrolases, to initiate the breakdown of pesticide molecules.^92^ The presence of multiple plasmid-borne degradative operons further enhances their adaptability and allows for the simultaneous degradation of complex pesticide mixtures (Table S1).

The *Sphingomonas* is another prominent group known for its role in the degradation of aromatic pesticides. *Sphingomonas spp.* have been implicated in the catabolism of herbicides such as atrazine and insecticides like carbofuran, employing monooxygenases and dioxygenases that cleave the aromatic rings, rendering the molecules more susceptible to further metabolic processing.^93^ The unique glycosphingolipid-rich cell membranes of *Sphingomonas spp.* are believed to confer enhanced resistance to hydrophobic pesticides, facilitating their survival and activity in contaminated environments.^94^

Members of the *Bacillus* have also demonstrated significant potential in pesticide bioremediation.^95^ *Bacillus subtilis* and *Bacillus thuringiensis* strains are known for their production of extracellular hydrolytic enzymes that degrade ester and amide bonds commonly found in pesticides. Moreover, *Bacillus species* can form endospores, enabling them to withstand adverse environmental conditions and thus maintain their degradative capabilities over extended periods.^96^ The resilience and robustness of *Bacillus spp.* make them suitable candidates for bioaugmentation strategies in field-scale bioremediation projects.

Actinomycetes, particularly species belonging to the *Streptomyces*, contribute substantially to the degradation of persistent organic pollutants, including chlorinated pesticides such as DDT and Hexachlorocyclohexane (HCH).^97^ *Streptomyces spp.* produces an extensive array of secondary metabolites and enzymes, including dehalogenases and oxygenases, which facilitate the breakdown of complex pesticide molecules.^98^ Their filamentous morphology and capacity to form extensive mycelial networks in soil environments enhance their access to hydrophobic pesticide residues that are otherwise poorly bioavailable.

Fungi play a pivotal role in pesticide biotransformation, especially in terrestrial ecosystems. White-rot fungi, such as *Phanerochaete chrysosporium* and Trametes versicolor, are renowned for their ligninolytic enzyme systems comprising lignin peroxidase, manganese peroxidase, and laccase.^99^ These enzymes exhibit substrate specificity and can oxidize a wide range of structurally diverse pesticides, including organophosphates, carbamates, and polycyclic aromatic hydrocarbons.^100^ Fungal degradation mechanisms are primarily oxidative and are facilitated by the secretion of extracellular enzymes, allowing fungi to act on insoluble or recalcitrant pesticide residues.

*Aspergillus* and *Penicillium* species, widely distributed soil fungi, have also demonstrated abilities to degrade various classes of pesticides through intracellular enzymatic systems.^101^ These fungi employ cytochrome P450 monooxygenases and hydrolases to catalyze the initial transformations of pesticide molecules, followed by conjugation and sequestration of metabolites. The adaptability of these fungi to diverse environmental conditions and their high enzyme production capacity makes them valuable agents for bioremediation applications. Although less extensively studied, archaea are increasingly recognized for their role in the degradation of environmental contaminants, including pesticides. Certain extremophilic archaea, particularly members of the genera *Halobacterium* (phylum Euryarchaeota) from hypersaline environments, *Sulfolobus* (phylum Crenarchaeota) from acidic hot springs, and *Thermococcus* (phylum Euryarchaeota) from hydrothermal vents, have exhibited enzymatic activities that contribute to the breakdown of organophosphorus esters and other xenobiotics.^102^ The unique membrane lipid composition and enzyme systems of archaea confer resistance to harsh environmental conditions, positioning them as promising candidates for bioremediation in extreme environments where conventional microbial systems may fail.^103^

The success of microbial pesticide biotransformation is not solely dependent on individual taxa but often results from synergistic interactions within microbial communities. Consortia comprising bacteria, and fungi can collectively degrade complex pesticide mixtures more efficiently than individual strains due to metabolic cooperation and division of labor. In these consortia, primary degraders initiate the breakdown of pesticide molecules, producing intermediate metabolites that are subsequently utilized by secondary degraders, leading to complete mineralization.^92^ Such synergistic interactions are critical in natural environments where pesticide contamination often involves mixtures of compounds with varying chemical structures and degradation pathways. The identification and characterization of key microbial taxa involved in pesticide biotransformation have been greatly facilitated by the advent of culture-independent molecular techniques, including 16S rRNA gene sequencing, metagenomics, and meta-transcriptomics. These tools have uncovered a greater diversity of pesticide-degrading microorganisms than previously recognized and have provided insights into the ecological dynamics and functional potential of microbial communities in contaminated environments.^77^ Biotransformation of pesticides is mediated by a diverse assemblage of microbial taxa, each contributing distinct enzymatic capabilities and ecological strategies. Understanding the taxonomy, physiology, and metabolic pathways of these microorganisms is essential for the development of effective bioremediation strategies and for predicting the environmental fate of pesticide residues.

## 5 Animal and Human Biotransformation of Pesticides

The metabolism of pesticides in animals and humans plays a crucial role in determining the toxico-kinetics, bioavailability, and ultimate toxicity of these compounds. Biotransformation processes are essential for the detoxification and elimination of xenobiotic substances, including pesticides, and involve complex enzymatic pathways that modulate their biological activity. However, in certain cases, metabolic activation can lead to the formation of more toxic or reactive intermediates, posing significant risks to human health and the environment. Understanding the enzymatic and molecular mechanisms underlying pesticide biotransformation in animal systems is fundamental for risk assessment, regulatory decisions, and the development of intervention strategies aimed at mitigating pesticide-related toxicities. Pesticide metabolism in animals and humans is typically conceptualized in terms of two sequential phases. Phase I reactions, or functionalization reactions, involve the introduction or exposure of functional groups on the pesticide molecule, rendering it more polar and reactive. Phase II reactions, or conjugation reactions, involve the covalent attachment of endogenous hydrophilic molecules to the functionalized pesticide or its metabolites, enhancing their solubility and facilitating excretion. In some cases, a third phase, involving the active transport of conjugated metabolites out of cells, is also recognized.^104^ The liver serves as the primary site of pesticide biotransformation in mammals, owing to its high concentration of detoxifying enzymes and its central role in xenobiotic metabolism. Nonetheless, other tissues such as the kidneys, lungs, gastrointestinal tract, and skin also contribute to biotransformation processes, albeit to a lesser extent.^105^ Phase I reactions are predominantly catalyzed by the cytochrome P450 monooxygenase (CYP450) enzyme system, a superfamily of heme-containing enzymes capable of catalyzing a diverse array of oxidative reactions, including hydroxylation, epoxidation, dealkylation, and deamination. CYP450 enzymes are classified into multiple families and subfamilies based on sequence homology, with CYP1, CYP2, and CYP3 families being most relevant to pesticide metabolism in humans.^106^ CYP2B6 and CYP3A4 are major enzymes involved in the oxidation of organophosphate pesticides such as chlorpyrifos and parathion, converting them into their respective oxon metabolites, which are potent inhibitors of acetylcholinesterase and responsible for the acute neurotoxicity associated with organophosphate poisoning.^20^

Genetic polymorphisms in detoxification enzymes further contribute to interindividual variability in pesticide metabolism and toxicity in human populations. Polymorphisms in genes encoding CYP450 enzymes, GSTs, and UGTs can result in altered enzyme activity, influencing the rate of pesticide biotransformation and the risk of adverse health effects.^107^ Individuals with null variants of GSTM1 and GSTT1 genes may have impaired detoxification capacity, increasing their vulnerability to pesticide-induced oxidative stress and related pathologies.

Biomonitoring of pesticide exposure and metabolism in humans often involves the measurement of specific metabolites in biological matrices such as blood, urine, and hair. The urinary metabolite 3,5,6-trichloro-2-pyridinol (TCPy) is a biomarker for exposure to chlorpyrifos and related organophosphates.^108^ Advances in analytical techniques, including liquid chromatography-tandem mass spectrometry (LC-MS/MS) and high-resolution mass spectrometry (HRMS), have enhanced the sensitivity and specificity of biomarker detection, enabling more accurate exposure assessment and epidemiological studies.^109^

The toxico-kinetics of pesticide biotransformation encompass absorption, distribution, metabolism, and excretion (ADME) processes. Pesticides can enter the body through ingestion, inhalation, or dermal absorption, with lipophilic compounds readily crossing biological membranes. Following absorption, pesticides distribute throughout the body, often accumulating in lipid-rich tissues such as adipose tissue and nervous system. Metabolism serves to transform these compounds into more hydrophilic metabolites, which are excreted primarily via the kidneys or biliary system. The half-life of pesticide in the body is determined by the efficiency of these processes and has implications for the duration and severity of exposure.^110^ Dose-dependent effects play a critical role in shaping pesticide metabolism in animals and humans. For organophosphates such as chlorpyrifos, physiologically based pharmacokinetic/pharmacodynamic (PBPK/PD) models demonstrate nonlinear oxon formation at higher exposures, reflecting a balance between CYP-mediated activation and detoxification routes such as paraoxonase-1 (PON1) and esterases.^111^ As doses increase, these protective pathways can saturate, leading to disproportionate rises in toxic metabolites and acetylcholinesterase inhibition. More broadly, conjugation pathways including sulfation and glucuronidation often saturate at elevated exposure levels, shifting the metabolic flux toward oxidative reactions and the generation of reactive intermediates. This dose-dependent interplay, illustrated in organophosphates, pyrethroids, and phenolic pesticide metabolites, underscores the importance of considering exposure intensity when evaluating toxicological outcomes and interindividual variability in risk assessment.^112^

Understanding the metabolic fate of pesticides in animals and humans is essential for the assessment of health risks associated with pesticide exposure. Regulatory agencies rely on metabolic data to establish acceptable daily intakes (ADIs) and maximum residue limits (MRLs) for pesticides in food and environmental media.^103^ Furthermore, insights into metabolic pathways inform the development of therapeutic interventions for pesticide poisoning, such as the use of atropine and pralidoxime for organophosphate toxicity. The biotransformation of pesticides in animals and humans is a complex, enzyme-mediated process that modulates the toxicity and persistence of these compounds. While detoxification is the primary outcome, the potential for bioactivation underscores the need for comprehensive risk assessments that consider both parent compounds and their metabolites. Continued research into the molecular mechanisms of pesticide metabolism, interspecies differences, and the influence of genetic polymorphisms is crucial for advancing public health protections and environmental safety.

## 6 Analytical Advances in Studying Pesticide Biotransformation

Understanding the complex biochemical pathways and environmental behavior of pesticides and their transformation products necessitates the use of advanced analytical methodologies. Traditional chemical analyses, while effective for detecting parent pesticides, often fall short in identifying the diverse array of metabolites produced during biotransformation. Recent advancements in analytical chemistry, coupled with the integration of omics technologies, have significantly enhanced the capacity to characterize both known and unknown transformation products, decipher metabolic pathways, and quantify trace-level residues across complex biological and environmental matrices.^114^ The study of pesticide biotransformation fundamentally relies on the ability to detect, identify, and quantify both parent compounds and their metabolites. Analytical techniques must therefore offer high sensitivity, selectivity, and resolution, particularly given the often-low environmental concentrations and the structural similarity of many transformation products. Mass spectrometry (MS)-based techniques, in combination with chromatographic separation methods, have become the cornerstone of modern pesticide analysis.^115^

High-performance liquid chromatography (HPLC) coupled with tandem mass spectrometry (LC-MS/MS) has emerged as a leading platform for pesticide residue analysis. This technique offers high sensitivity and selectivity, enabling the quantification of pesticides and their metabolites at parts-per-trillion levels in complex matrices such as soil, water, food, and biological tissues.^116^ The soft ionization provided by electrospray ionization (ESI) or atmospheric pressure chemical ionization (APCI) in LC-MS/MS minimizes fragmentation, preserving the molecular ion and facilitating accurate mass determination.^117^ Advances in ultrahigh-performance liquid chromatography (UHPLC) have further improved chromatographic resolution and reduced analysis times, enhancing the throughput of multiresidue pesticide monitoring programs.

Gas chromatography coupled with mass spectrometry (GC-MS) remains an important analytical tool, particularly for volatile and semivolatile pesticides. GC-MS methods are often employed for the analysis of organochlorine pesticides, pyrethroids, and volatile organophosphates.^118,119^ Derivatization techniques can be applied to enhance the volatility and stability of polar metabolites, expanding the applicability of GC-MS to a wider range of analytes. The advent of tandem mass spectrometry (GC-MS/MS) has further enhanced the selectivity and sensitivity of GC-based analyses, enabling the detection of trace-level pesticide residues with minimal matrix interference.^120^

High-resolution mass spectrometry (HRMS) techniques, including time-of-flight (TOF) and orbitrap-based instruments, have revolutionized the field by enabling nontargeted screening and the elucidation of unknown metabolites.^121^ HRMS offers accurate mass measurements with parts-per-million (ppm) mass accuracy, facilitating the identification of unknown compounds through molecular formula determination and isotope pattern analysis. Data-independent acquisition (DIA) and data-dependent acquisition (DDA) workflows in HRMS allow for comprehensive sample profiling, capturing both known and unknown analytes in a single analysis.^122^ Complementary to MS-based techniques, nuclear magnetic resonance (NMR) spectroscopy provides detailed structural information on pesticide metabolites. Although less sensitive than MS, NMR is unparalleled in its ability to elucidate molecular structures, particularly in the characterization of stereochemistry and functional group positioning.^123^ Advances in cryoprobe technology and multidimensional NMR techniques have enhanced the sensitivity and resolution of NMR spectroscopy, enabling the characterization of metabolites present at low concentrations.

Stable isotope probing (SIP) represents a powerful technique for linking biotransformation processes to specific microbial taxa.^124^ In SIP experiments, isotopically labeled pesticides (e.g., 13C- or 15N-labeled compounds) are introduced into environmental samples, and the incorporation of the label into microbial biomolecules such as DNA, RNA, or phospholipid fatty acids (PLFAs) is tracked. Subsequent analysis of labeled biomolecules using MS or NMR allows for the identification of active pesticide degraders and the elucidation of metabolic pathways.^125^ SIP has provided critical insights into the microbial ecology of pesticide degradation, revealing previously unrecognized degraders and metabolic routes.

Metabolomics, the comprehensive analysis of small-molecule metabolites, has become an indispensable tool in pesticide biotransformation studies. Metabolomic approaches can be targeted, focusing on known metabolites, or untargeted, aiming to capture the entire spectrum of metabolic changes induced by pesticide exposure. High-resolution MS and NMR are the primary analytical platforms for metabolomics, with multivariate statistical analyses such as principal component analysis (PCA) and partial least-squares-discriminant analysis (PLS-DA) employed to interpret complex data sets. Metabolomic profiling enables the identification of biomarkers of pesticide exposure and toxicity, as well as the elucidation of novel degradation pathways.^126^

The integration of omics technologies, including genomics, transcriptomics, proteomics, and metabolomics, into multiomics approaches has further expanded the analytical toolbox available for studying pesticide biotransformation. Multiomics integration allows for the simultaneous assessment of gene expression, protein abundance, and metabolite profiles, providing a holistic view of the biological responses to pesticide exposure. Systems biology frameworks, incorporating network analysis and pathway modeling, facilitate the identification of key regulatory nodes and interactions governing pesticide metabolism.

Emerging technologies such as matrix-assisted laser desorption/ionization imaging mass spectrometry (MALDI-IMS) and laser ablation electrospray ionization mass spectrometry (LAESI-MS) offer spatially resolved analyses, enabling the visualization of pesticide and metabolite distributions within biological tissues.^127^ These techniques provide critical insights into the localization and compartmentalization of biotransformation processes at the tissue and cellular levels.

Multiomics approaches have begun to reveal how microbial degraders orchestrate pesticide transformation at the systems level. In Achromobacter xylosoxidans SL-6, combined transcriptomic and metabolomic profiling of diuron degradation mapped sequential transformations including urea-bridge cleavage, dehalogenation, deamination, and aromatic ring opening to cis, cis-muconic acid.^128^ These molecular data not only reconstructed the degradation pathway but also highlighted induction of stress-response genes and antioxidant systems that underpin microbial adaptation to xenobiotic pressure.^129^ At the community scale, metagenomics and metabolic modeling of atrazine-exposed soils uncovered interdependencies between degrader and nondegrader taxa, showing how metabolic cooperation sustains pesticide mineralization. Such insights have guided the design of biostimulation strategies that favor key functional guilds in agricultural soils.^129^

A rhizosphere perspective further illustrates the power of multiomics integration. In crop–soil systems exposed to herbicides, coordinated metagenomic and metabolomic surveys captured shifts in microbial community structure alongside distinct metabolite profiles, linking pesticide exposure to altered plant–microbiome interactions. This study provided an insight how host plants and their associated microbiota jointly mediate degradation processes and mitigate phytotoxic stress. This study demonstrates that multiomics approaches can connect genes to enzymes, metabolites, and ecological function, enabling a mechanistic and predictive understanding of pesticide biotransformation.^130^

Synthetic biology approaches build upon omics insights by enabling the rational design of microorganisms and plants with improved pesticide-degrading capacity. For instance, *Escherichia coli* has been engineered to coexpress an organophosphate hydrolase gene (mpd) and an organochlorine-degrading gene (linA), allowing the bacterium to break down multiple pesticide classes simultaneously.^131^ Biosafety strategies such as “suicide gene” circuits have also been introduced into engineered degraders to ensure containment under natural conditions. Moreover, synthetic microbial consortia have been assembled to coordinate complementary metabolic functions, achieving more efficient pesticide mineralization in complex environments.

Despite these advancements, challenges remain in the analytical characterization of pesticide biotransformation. The complexity of environmental and biological matrices can introduce significant matrix effects, impacting analytical sensitivity and accuracy. Furthermore, the identification of unknown metabolites often requires extensive analytical validation and structural confirmation, underscoring the need for comprehensive spectral databases and advanced data processing algorithms. Efforts to address these challenges include the development of reference libraries and databases such as METLIN, HMDB, and MassBank, which provide curated spectral information for a wide range of compounds, including pesticides and their metabolites.^132^ Machine learning and artificial intelligence approaches are also being explored for the automated interpretation of complex mass spectrometric and spectroscopic data, offering new avenues for the rapid and accurate identification of biotransformation products.^133^

The analytical landscape for studying pesticide biotransformation has evolved dramatically, driven by advancements in MS and NMR technologies, stable isotope techniques, and the integration of omics platforms. These tools have expanded the ability to detect, identify, and quantify both parent compounds and metabolites, providing critical insights into the mechanisms, pathways, and ecological impacts of pesticide biotransformation. Continued innovation and methodological refinement will be essential for advancing the understanding of pesticide fate and for informing risk assessments and remediation strategies.

## 7 Transformation Products of Pesticides: Occurrence, Environmental Behavior, and Risk Assessment

The biotransformation of pesticides does not always culminate in complete mineralization to innocuous end products such as carbon dioxide and water.^134^ Rather, many degradation processes yield a range of intermediate and final transformation products (TPs), often referred to as metabolites, which can exhibit chemical structures and properties distinct from their parent compounds.^135^ These transformation products can persist in environmental matrices, bioaccumulate in food webs, and in some cases, display equal or greater toxicity compared to the original pesticide. The occurrence, environmental behavior, and toxicological profiles of these TPs are of paramount importance for comprehensive environmental risk assessment and for the design of effective regulatory frameworks.

Transformation products can arise from a wide array of biotic and abiotic degradation processes. Biotic transformations are typically mediated by microorganisms, plants, or animal metabolic systems, involving enzymatic reactions such as oxidation, reduction, hydrolysis, and conjugation. Abiotic transformations can occur via photolysis, hydrolysis, and chemical oxidation in the environment.^136^ In both cases, the resulting TPs can differ markedly in terms of polarity, solubility, volatility, and persistence.

One notable example of a transformation product of significant environmental concern is 3,5,6-trichloro-2-pyridinol (TCPy), formed during the microbial and animal metabolism of chlorpyrifos, a widely used organophosphate insecticide. Although TCPy is less acutely toxic than chlorpyrifos itself, it is highly persistent, water-soluble, and has been detected in groundwater, surface water, and human biological samples. Its widespread occurrence underscores the necessity of monitoring not just parent compounds but also their metabolites in environmental matrices. The degradation of atrazine, a triazine herbicide, results in several major transformation products, including desethylatrazine (DEA) and desisopropylatrazine (DIA).^137^ These TPs are more polar than atrazine, enhancing their mobility in soil and groundwater systems. DEA and DIA have been frequently detected in drinking water supplies at concentrations exceeding regulatory limits, raising concerns about chronic exposure and associated health risks. The environmental behavior of transformation products is governed by their physicochemical properties, including water solubility, vapor pressure, octanol–water partition coefficient (*K*_ow_), and degradation half-life (DT_50_).^138^ Polar metabolites generally exhibit increased mobility in the environment, enhancing their potential for groundwater contamination. Conversely, nonpolar metabolites may preferentially partition into sediments and biota, contributing to bioaccumulation and biomagnification in food webs. From a toxicological perspective, transformation products can exhibit a range of activities, including endocrine disruption, genotoxicity, and neurotoxicity. Some TPs are biologically inert and of negligible concern, but others may retain or even surpass the biological activity of the parent compound. For instance, the oxidative metabolism of organophosphorus pesticides often yields oxon derivatives that are more potent as acetylcholinesterase inhibitors than the original phosphorothioate compounds.^21^ Similarly, certain breakdown products of neonicotinoid insecticides have been shown to possess high affinity for nicotinic acetylcholine receptors, raising concerns about their impacts on nontarget organisms, particularly pollinators.

In aquatic environments, the photolytic degradation of pesticides can produce TPs with altered ecological effects. Photo transformation of fipronil, an insecticide commonly used in urban pest control, yields sulfone and desulfinyl derivatives that are more persistent and equally toxic to aquatic invertebrates.^139^ These findings highlight the complexity of assessing the environmental risks of pesticide use, as the fate and effects of TPs must be considered alongside those of parent compounds.

The detection and identification of transformation products present significant analytical challenges. TPs are often present at lower concentrations than parent compounds and may lack characteristic spectral signatures, complicating their identification using conventional targeted analytical methods. Advances in high-resolution mass spectrometry (HRMS), particularly nontargeted screening approaches, have greatly enhanced the ability to detect and elucidate unknown TPs. Coupled with computational tools for structural elucidation and predictive modeling, these technologies have expanded the scope of environmental monitoring and facilitated a more comprehensive understanding of pesticide fate.

Risk assessment frameworks have evolved to incorporate transformation products, recognizing their potential contribution to overall exposure and risk. The European Union’s REACH regulation and the United States Environmental Protection Agency (USEPA) guidelines now require the evaluation of TPs during pesticide registration processes.^113,140^ However, gaps remain in the toxicological characterization of many TPs, and regulatory thresholds for their presence in environmental media are often lacking or inconsistently applied.

To address these gaps, a tiered approach to TP risk assessment has been proposed, encompassing initial screening based on structural similarity to known toxicants, followed by targeted toxicological testing for high-priority metabolites. Emerging tools such as quantitative structure–activity relationship (QSAR) modeling and in vitro bioassays offer cost-effective means for prioritizing TPs for further study.^141^ Another critical consideration is the cumulative and synergistic effects of pesticide mixtures and their TPs. In real-world scenarios, organisms are often exposed to complex mixtures of chemicals, and additive or synergistic interactions can exacerbate toxic outcomes. Mixture toxicity models and cumulative risk assessment frameworks are being developed to better account for these interactions. The persistence of certain transformation products also has implications for long-term environmental contamination. Persistent TPs can accumulate in sediments and groundwater reservoirs, acting as secondary sources of pollution even after the cessation of pesticide use. For instance, persistent transformation products of DDT, such as DDE and DDD, continue to be detected in aquatic ecosystems decades after the banning of DDT.^142^ In the context of climate change, alterations in temperature, precipitation patterns, and extreme weather events are expected to influence the degradation kinetics and mobility of both pesticides and their TPs. Warmer temperatures may accelerate certain biotic and abiotic transformation processes, while increased precipitation could enhance leaching and runoff, leading to greater dissemination of TPs in the environment. Transformation products of pesticides represent a critical, yet often underappreciated, dimension of environmental contamination and toxicological risk. Comprehensive evaluation of TPs requires advanced analytical techniques, robust risk assessment methodologies, and a holistic understanding of environmental fate processes. Future research must prioritize the identification and toxicological characterization of TPs, the elucidation of their environmental behavior under changing climatic conditions, and the development of regulatory frameworks that adequately address their risks.

Recent advances in computational biology provide powerful tools to complement experimental studies of pesticide metabolism.^143^ Molecular docking and molecular dynamics (MD) simulations are increasingly used to predict pesticide binding within detoxification enzymes such as cytochrome P450s, esterases, and laccases. Docking scores and substrate orientations relative to catalytic residues often correlate with observed metabolism rates, while MD simulations capture enzyme flexibility and dynamic active-site interactions. For example, docking analyses of neonicotinoids in insect P450s have identified metabolic hot-spots that explain differences between susceptible and resistant populations. Beyond structure-based modeling, machine learning (ML) and quantitative structure–activity relationship (QSAR) models are emerging as predictive frameworks for pesticide biotransformation.^144,145^ Tools such as XenoBug and DeepEC leverage large enzyme–substrate data sets and metagenomic sequence repositories to identify novel biodegradative enzymes and predict transformation pathways.^146,147^ These approaches enable in silico screening of candidate degraders, guiding enzyme engineering and reducing reliance on trial-and-error experimentation.

## 8 Integrated Remediation Approaches for Pesticide-Contaminated Environments

The widespread use and persistence of pesticides in agricultural, urban, and industrial environments have resulted in significant contamination of soils, water bodies, and biota. Conventional remediation techniques, including physical removal, chemical oxidation, and containment, often fall short in addressing the complexity of pesticide pollution, particularly the heterogeneous nature of contaminated matrices and the diverse chemical properties of pesticides and their transformation products. Consequently, integrated remediation approaches, combining multiple strategies and leveraging the synergistic potential of biological, chemical, and physical processes, have emerged as a more effective and sustainable solution for the remediation of pesticide-contaminated environments.

Bioremediation remains a cornerstone of integrated approaches, harnessing the metabolic capabilities of microorganisms and plants to transform and detoxify pesticides. Microbial bioremediation strategies encompass natural attenuation, biostimulation, and bioaugmentation.^148^ Natural attenuation relies on indigenous microbial communities to degrade contaminants without human intervention. However, the efficiency of natural attenuation is often limited by environmental factors such as nutrient availability, oxygen levels, and microbial community structure.^149^ To enhance bioremediation efficacy, biostimulation techniques involve the amendment of contaminated sites with nutrients (e.g., nitrogen, phosphorus) or electron donors/acceptors to stimulate the growth and activity of native degraders.^150^ Bioaugmentation introduces exogenous microbial strains or consortia with superior degradative capabilities into the contaminated site. This strategy has been successfully applied for the degradation of persistent pesticides such as atrazine and lindane, although challenges related to microbial survival, competition, and gene transfer must be carefully managed. Phytoremediation, the use of plants to remove, contain, or degrade contaminants, offers a cost-effective and environmentally friendly alternative or complement to microbial bioremediation. Phytoremediation strategies include phytoextraction, phytodegradation, phytostabilization, and rhizodegradation.^151^ In phytoextraction, plants uptake and accumulate pesticides in their biomass, which can then be harvested and properly disposed of. Phytodegradation involves the enzymatic breakdown of pesticides within plant tissues, often facilitated by cytochrome P450 monooxygenases, glutathione S-transferases, and other detoxification enzymes. Rhizodegradation, also known as phytostimulation, leverages the rhizosphere effect, where root exudates enhance microbial activity and diversity in the soil, promoting the microbial degradation of pesticides. This approach capitalizes on the synergistic interactions between plants and soil microbiota, enhancing the biodegradation process and improving soil health.

Chemical remediation techniques, including advanced oxidation processes (AOPs), are often integrated with bioremediation to achieve complete mineralization of pesticides and their recalcitrant transformation products. AOPs, such as Fenton’s reagent, ozonation, and photocatalysis, generate highly reactive hydroxyl radicals capable of nonselective oxidation of organic pollutants.^152^ While AOPs can achieve rapid degradation, they are energy-intensive and may produce toxic byproducts if not properly optimized. Thus, coupling AOPs with bioremediation allows for the partial oxidation of complex pesticide molecules into more biodegradable intermediates, subsequently mineralized by microbial consortia in a process known as bio-oxidation coupling.

Constructed wetlands represent another integrated remediation strategy, combining physical, chemical, and biological processes to remove pesticides from contaminated water. Wetland systems utilize sedimentation, filtration, plant uptake, microbial degradation, and photolysis to achieve contaminant removal.^153^ Studies have demonstrated the effective removal of herbicides such as atrazine and simazine in constructed wetland systems, highlighting their potential for agricultural runoff management.^154^

Recent advances in nanotechnology have introduced novel materials, such as nanoadsorbents and nanocatalysts, into the remediation toolbox. Engineered nanoparticles, including zerovalent iron (nZVI) and titanium dioxide (TiO_2_), exhibit high surface area and reactivity, enhancing the adsorption and degradation of pesticides.^155^ The integration of nanotechnology with biological systems, termed nanobioremediation, offers promising avenues for improving the efficiency and specificity of pesticide remediation efforts.

Electrokinetic remediation, involving the application of an electric field across contaminated soils, has been investigated as a means to enhance the mobility and bioavailability of pesticides, facilitating their removal or degradation. Electrokinetic processes can promote the transport of nutrients and electron acceptors, stimulating microbial activity and enhancing bioremediation outcomes.^156^ However, challenges related to energy consumption and potential soil disturbance must be addressed for large-scale applications.

Integrated remediation strategies must also consider site-specific conditions, including soil type, contaminant properties, climatic factors, and land use. The selection and design of appropriate remediation technologies requires comprehensive site assessments and feasibility studies, integrating chemical, biological, and ecological data. The application of decision support tools, such as multicriteria analysis and life cycle assessment, can aid in evaluating the environmental, economic, and social implications of different remediation options, facilitating the selection of sustainable and effective strategies.^157^ The role of regulatory frameworks and public policy is critical in promoting the adoption of integrated remediation approaches. Regulations must incentivize sustainable practices and provide guidelines for the safe and effective implementation of bioremediation technologies. Public-private partnerships, stakeholder engagement, and community involvement are essential for the successful deployment and long-term sustainability of remediation projects. The remediation of pesticide-contaminated environments requires integrated approaches that synergistically combine biological, chemical, and physical processes. The development and optimization of such strategies necessitate interdisciplinary research, technological innovation, and supportive policy frameworks. Future efforts should focus on enhancing the efficacy, scalability, and sustainability of integrated remediation technologies, ultimately contributing to the restoration of contaminated ecosystems and the protection of public health.

## 9 Conclusion

The biotransformation of pesticides by microorganisms, plants, and animals represents a pivotal process governing the fate of these chemicals in the environment and their impact on ecosystems and human health. Across microbial systems, plants, and animal models, a myriad of enzymatic pathways facilitates the conversion of persistent and often hydrophobic pesticide molecules into more hydrophilic and, ideally, less toxic derivatives. However, the complexity of these biotransformation processes, coupled with the formation of potentially hazardous transformation products, underscores the necessity for comprehensive scientific inquiry and robust regulatory oversight. Advances in analytical technologies, particularly high-resolution mass spectrometry, nuclear magnetic resonance spectroscopy, and integrated omics approaches, have substantially enhanced the capacity to elucidate biotransformation pathways and identify both known and novel transformation products. These technological developments have not only deepened the understanding of pesticide metabolism at a molecular level but have also revealed the ubiquity and persistence of pesticide residues and their metabolites in environmental matrices. Moreover, the integration of synthetic biology and nanotechnology into bioremediation strategies offers unprecedented opportunities for enhancing the degradation and removal of pesticides from contaminated environments. Despite these scientific advances, significant challenges remain. The environmental behavior and toxicological profiles of many transformation products are still poorly understood. Furthermore, the ecological risks associated with complex mixtures of pesticides and their metabolites, as well as their cumulative and synergistic effects, require further investigation. Climate change adds another layer of complexity, potentially altering degradation kinetics, bioavailability, and exposure pathways of pesticides and their transformation products. Policy and regulatory frameworks must evolve to address these emerging challenges. Current pesticide regulations, both in the European Union under REACH and in the United States under the Federal Insecticide, Fungicide, and Rodenticide Act (FIFRA), have made strides toward considering transformation products in risk assessments.^113,158^ However, more comprehensive and harmonized global policies are required to systematically evaluate the environmental fate and toxicity of transformation products. Regulatory agencies must incorporate advanced analytical techniques into monitoring programs and establish guidelines for acceptable levels of not only parent pesticides but also their metabolites in environmental and biological matrices. Furthermore, the adoption of more stringent requirements for environmental persistence, bioaccumulation potential, and toxicity of transformation products during pesticide approval processes is essential. Enhanced postapproval monitoring, coupled with transparent data sharing and public accessibility to monitoring results, will be critical in fostering public trust and ensuring environmental and human health protections. Future research must prioritize the development of predictive models for pesticide degradation pathways and transformation of product formation under varying environmental conditions. The integration of machine learning and artificial intelligence into environmental modeling holds promise for improving the prediction of degradation outcomes and informing risk assessments.^159^ Additionally, interdisciplinary collaborations that bridge environmental science, toxicology, agronomy, and socioeconomics are necessary to design sustainable pesticide use strategies and effective remediation approaches. The promotion of integrated pest management (IPM) strategies and the development of less persistent and more environmentally benign pesticides should be encouraged to reduce the reliance on chemical pesticides. Advances in biotechnology, such as RNA interference-based pest control and biologically derived pesticides, offer potential alternatives that align with the principles of sustainable agriculture and environmental stewardship. The sustainable management of pesticide use and the remediation of contaminated environments require a multifaceted strategy that integrates scientific innovation, regulatory reform, and societal engagement. Advances in the mechanistic understanding of pesticide biotransformation provide a foundation for more precise risk assessments, including the identification of transformation products with potential toxicological relevance, the evaluation of dose-dependent metabolic shifts, and the prediction of degradation outcomes under variable environmental conditions. Embedding these insights into regulatory frameworks, such as OECD environmental fate guidelines, U.S. EPA registration requirements, and EU metabolite relevance assessments can improve the scientific basis of pesticide approval and monitoring programs. Furthermore, incorporating multiomics approaches and computational biology into environmental policy could enable earlier detection of hazardous metabolites and more accurate modeling of long-term ecological risks. By bridging mechanistic science with policy and practice, pesticide biotransformation research can contribute to mitigating adverse environmental and human health impacts while fostering a more sustainable and resilient agricultural future. Despite progress in pesticide biotransformation research, critical gaps remain. The toxicological significance of transformation products is often poorly understood, with some metabolites potentially more persistent or hazardous than parent compounds. Dose-dependent shifts in metabolism are observed, yet quantitative thresholds across species and pesticide classes are lacking. Multiomics studies generate valuable insights, but integration into predictive models is still limited, and the impacts of pesticide mixtures and formulation additives remain underexplored. Translating laboratory findings into field-scale outcomes is challenged by environmental variability and ecological complexity. Addressing these gaps will require integrated approaches that combine high-resolution analytics, computational modeling, and long-term field validation to build a predictive, policy-relevant framework for sustainable pesticide management.

## Supplementary Material



## Notes

Summary of studies on pesticide biotransformation across biological systems (microbial, plant, animal, enzymatic, and integrated remediation approaches) (Table S1); representative plant cytochrome P450s (CYPs) and glutathione S-transferases (GSTs) involved in pesticide detoxification, with example substrates and reaction types (Table S2); conceptual pathway of organophosphate biotransformation (Figure S1); biotransformation pathway of neonicotinoid insecticides (Figure S2), and biotransformation pathway of triazine herbicides (Figure S3) (PDF)
