# Supplementary material for: Biotransformation of Pesticides across Biological Systems: Molecular Mechanisms, Omics Insights, and Biotechnological Advances for Environmental Sustainability
Source: ACS Omega. 2025 Oct 24;10(43):50709–23. doi: 10.1021/acsomega.5c06484 (PMC12592964; doi:10.1021/acsomega.5c06484)
Supplement: Supplementary file 1 [file ao5c06484_si_001.pdf]

Supporting information

Biotransformation of pesticides across biological systems: molecular mechanisms, omics insights, and biotechnological advances for environmental sustainability

Gayatri Basapuram[1], Avishek Dutta[1,2], Srimanti Duttagupta[1*]

1. Department of Geology, University of Georgia, Athens GA 30602, USA
2. Savannah River Ecology Laboratory, University of Georgia, Aiken, SC 29802, USA

**Corresponding author: Srimanti Duttagupta (sduttagupta@uga.edu)*

To synthesize the diverse literature on pesticide biotransformation, the previous studies were categorized according to the biological system or mechanism involved (microbial, plant, animal, enzymatic, or integrated remediation approaches). Table S1 summarizes the types of pesticides investigated, key references, and geographical contexts, highlighting both mechanistic insights and applied perspectives across biological systems.

Table S1: Studies on pesticide biotransformation across biological systems, categorized by the system or mechanism involved.

Mode of Biotransformation	Specific Pesticide(s)	References	Country / Location
**Microbial degradation – atrazine and triazines**	Atrazine, s-triazines	de Souza et al., 1998; Sadowsky, 2010; González Brambila et al., 2025	USA; Global; Mexico
**Microbial degradation – carbamates & organophosphates**	Carbofuran, malathion, carbamates, organophosphates	Chapalamadugu & Chaudhry, 1992; Malhotra et al., 2021; Kumar et al., 2019; Lestari et al., 2024	Global; India; Indonesia
**Microbial degradation – pyrethroids & HCH**	Fenpropathrin, HCH isomers	Chen et al., 2014; Cuozzo et al., 2018; Makarani & Kaushal, 2025	China; Argentina; Global
**Microbial consortia and community interactions**	Multiple pesticides / organic pollutants	Lü et al., 2024; Ray et al., 2024; Ahmad et al., 2022; Hussain et al., 2018; Roy et al., 2018; Varjani & Upasani, 2019; Banu et al., 2024; Zhang et al., 2017; Arif et al., 2012; Singh & Walker, 2006	China; India; USA; Argentina; Global

Mode of Biotransformation	Specific Pesticide(s)	References	Country / Location
**Enzymatic mechanisms – Phase I (activation, oxidation, hydrolysis)**	Organophosphates, carbamates, pyrethroids	Guengerich, 2001; Bhatt et al., 2021; Liu et al., 2015; Chen CH, 2024a; Pandian et al., 2020; Pandey et al., 2017; Chakraborty et al., 2023	USA; China; India; Japan
**Enzymatic mechanisms – Phase II (conjugation, detoxification)**	Imidacloprid, 2,4-D, dicamba, malathion	Salinas & Wong, 1999; Reinen & Vermeulen, 2015; Chen CH, 2024b; Chiu et al., 2018; Chen et al., 2019; Xu et al., 2025; Testa & Kraemer, 2008	Netherlands; USA; China; Global
**Plant metabolism ("green liver" concept, phytoremediation)**	Herbicides, xenobiotics (general), atrazine	Sandermann, 1999; Burken, 2003; Van Eerd et al., 2003; Schröder, 2007; Zhang & Yang, 2021; Cole, 1994; Vicidomini et al., 2024; Yu & Ahammed, 2025	Germany; USA; Canada; Italy; China
**Animal metabolism & toxicokinetics**	Malathion, aflatoxin B1, pesticides (general), 6PPD	Buratti et al., 2005; Eaton et al., 2025; Nyman et al., 2014; Dorne et al., 2005; Wormhoudt et al., 1999; Rao et al., 2025	Italy; USA; Switzerland; Netherlands; Global
**Fungal and actinobacterial biotransformation**	Organochlorines, organophosphates, lignocellulose-linked pollutants	Shanmugapriya et al., 2019; Cuozzo et al., 2018; Makarani & Kaushal, 2025; Zhuo & Fan, 2021; Matúš et al., 2023	India; Argentina; Global
**Advanced approaches – omics, metagenomics, bioengineering**	Multiple pesticides (general xenobiotics)	Fenner et al., 2021; Malik et al., 2021; Mishra et al., 2021; Ahmad et al., 2022; Zhang et al., 2017; Li et al., 2025; Jarmusch et al., 2021	Switzerland; India; USA; Global
**Biotransformation in aquatic/soil systems**	Pesticide residues in fish, birds, rice-fish, soil	Gaunt, 1996; Clasen et al., 2018; Kuo et al., 2022	USA; Brazil; Canada
**Bioremediation enhancement (bioaugmentation, stimulation, consortia)**	Oil sludge, pesticides, xenobiotics	Roy et al., 2018; Varjani & Upasani, 2019; Hussain et al., 2018; Ahmad et al., 2022; Lü et al., 2024	India; China; Global

Table S2: Representative plant cytochrome P450s (CYPs) and glutathione S-transferases (GSTs) involved in pesticide detoxification, with example substrates and reaction types

Enzyme family/class	Representative enzyme	Plant species	Example pesticide substrates	Reaction type (Phase I/II)
**Cytochrome P450s (CYPs)**	CYP71A subfamily	*Oryza sativa* (rice)	Phenylurea herbicides (linuron, diuron)	Hydroxylation, N-dealkylation (Phase I)
	CYP79A1	*Sorghum bicolor* (sorghum)	Atrazine (triazine herbicide)	Hydroxylation (Phase I)
	CYP76B1	*Helianthus annuus* (sunflower)	Phenylureas, triazines	Hydroxylation (Phase I)
	CYP81A6	*Oryza sativa* (rice)	Bentazon, sulfonylureas	Hydroxylation, detoxification (Phase I)
	CYP94C1	*Arabidopsis thaliana*	Herbicide safeners	ω-hydroxylation (Phase I, signaling-linked)
**Glutathione S-transferases (GSTs)**	GST Phi class (ZmGSTF1, ZmGSTF2)	*Zea mays* (maize)	Atrazine, alachlor	Glutathione conjugation (Phase II)
	GST Tau class (AtGSTU19, AtGSTU24)	*Arabidopsis thaliana*	Alachlor, atrazine, metolachlor	Glutathione conjugation (Phase II)
	GST Lambda class	*Triticum aestivum* (wheat)	Organochlorine metabolites	Redox reactions, conjugation (Phase II)
	GST Zeta class	*Arabidopsis thaliana*	Dichloroacetic acid (metabolite)	Dehalogenation (Phase II)

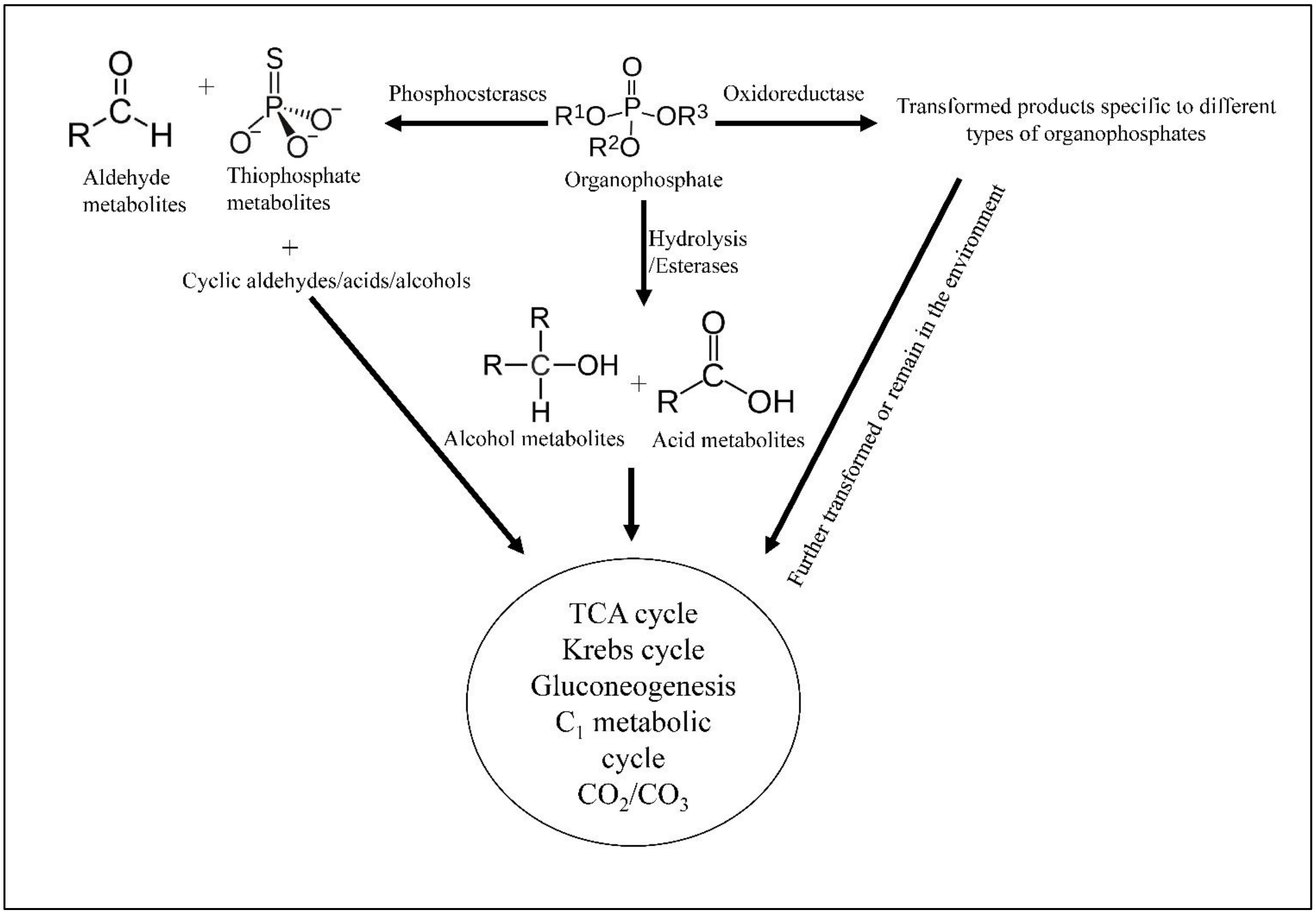

Figure S1: Conceptual pathway of organophosphate biotransformation

Organophosphate parent compounds undergo enzymatic transformation through multiple routes including hydrolysis by esterases/phosphoesterases, oxidation by oxidoreductases, and desulfuration/dealkylation. These processes yield a variety of metabolites such as alcohols, aldehydes, carboxylic acids, and thiophosphates, along with class-specific transformation products. Some intermediates may persist in the environment, while others enter central metabolic pathways such as the tricarboxylic acid (TCA) cycle, gluconeogenesis, and C_1 metabolism, ultimately leading to mineralization as CO_2 and H_2O.

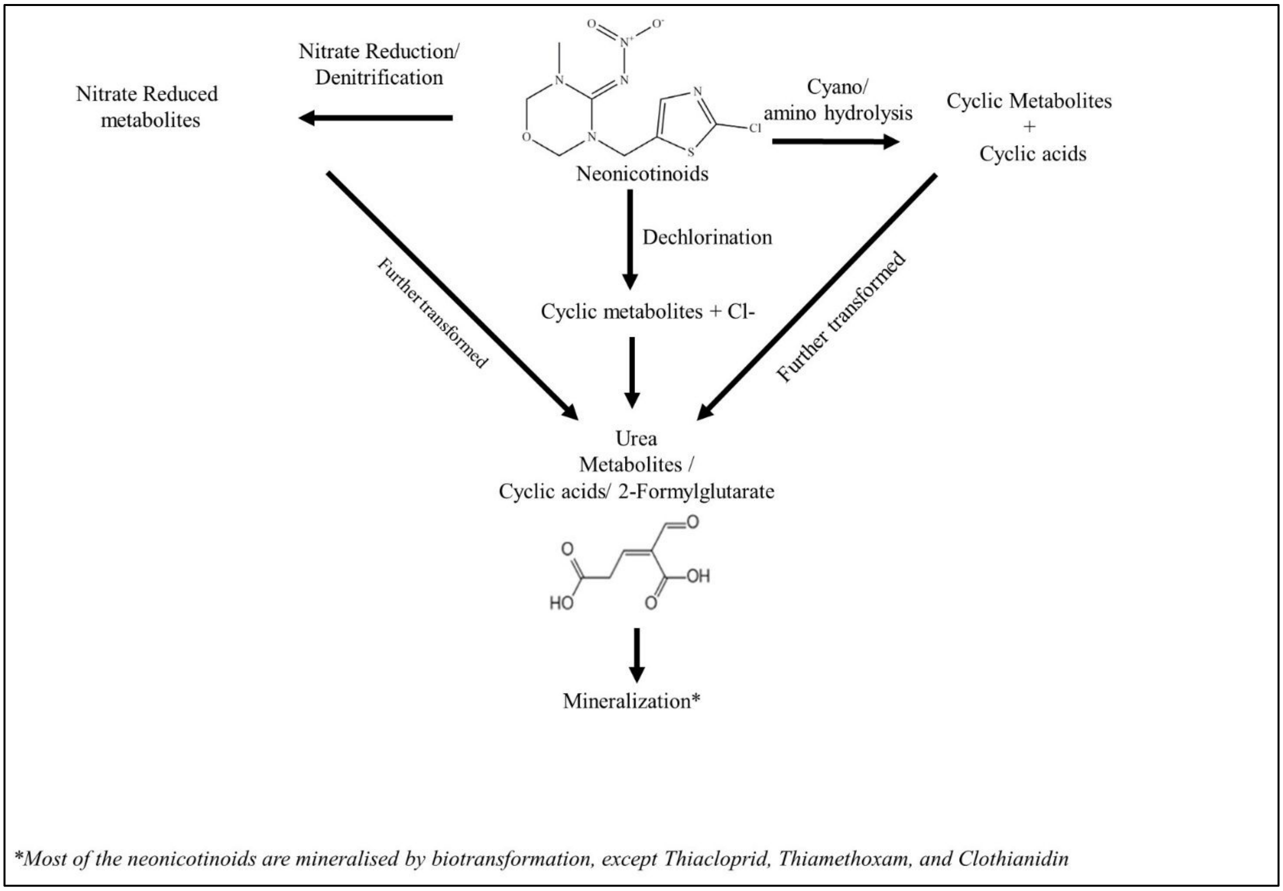

Figure S2: Biotransformation pathway of neonicotinoid insecticides

Neonicotinoids are transformed through multiple enzymatic reactions including nitrate reduction/denitrification, cyano/amino hydrolysis, and dechlorination. These processes generate nitrate-reduced metabolites, cyclic metabolites, urea derivatives, and dicarboxylic intermediates such as 2-formylglutarate. Further transformations ultimately lead to mineralization, although some neonicotinoids (e.g., thiacloprid, thiamethoxam, and clothianidin) show persistence and incomplete degradation. The scheme highlights the diversity of microbial routes contributing to neonicotinoid fate in the environment.

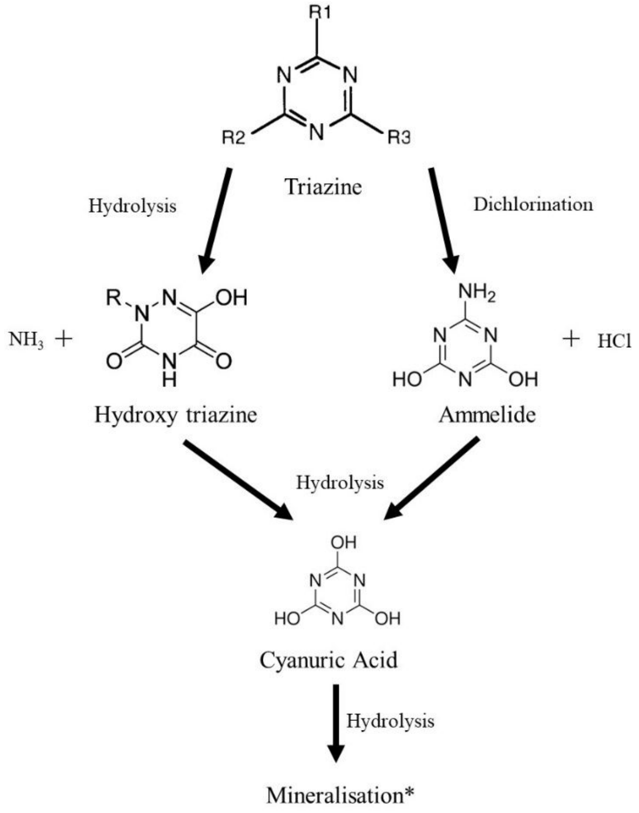

**Triazines are mineralised by biotransformation into ammonia, carbon dioxide and water*

Figure S3: Biotransformation pathway of triazine herbicides

Triazines undergo initial hydrolytic or dechlorination reactions leading to hydroxylated or dechlorinated intermediates such as hydroxytriazine and ammelide, with release of ammonia (NH_3) and hydrochloric acid (HCl). Subsequent hydrolysis yields cyanuric acid, which is further degraded to mineralization products including ammonia, carbon dioxide, and water. This pathway illustrates the sequential enzymatic steps responsible for the complete microbial breakdown of triazine herbicides.
